# Impact of Aortic Valve Calcium Burden on Paravalvular Leak Across Balloon-Expandable and Self-Expanding Valves: Insights from a Large TAVR Cohort

**DOI:** 10.3390/jcm15093239

**Published:** 2026-04-24

**Authors:** Ziad Arow, Laurent Lepage, Juri Iwata, Akiko Masumoto, Abid Assali, Mustafa Gabarin, Rawia Hussein-Aro, Chiara De Biase, Nicolas Dumonteil, Didier Tchetche, Laurent Bonfils

**Affiliations:** 1 Groupe CardioVasculaire Interventionnel, Clinique Pasteur, 31076 Toulouse, France; laulepage@yahoo.fr (L.L.); juriandbbpomecr@gmail.com (J.I.); akikomasu@gmail.com (A.M.); chiadebiase@gmail.com (C.D.B.); ndumonteil@clinique-pasteur.com (N.D.); dtchetche@clinique-pasteur.com (D.T.); lbonfils@clinique-pasteur.com (L.B.); 2 Cardiology Department, Meir Medical Center, Tel Aviv University, Kfar Saba 4410202, Israelmustafa.gabarin88@gmail.com (M.G.); 3 Hillel Yaffe Medical Center, Technion—Israel Institute of Technology, Haifa 3478403, Israel; rawiahu@gmail.com

**Keywords:** transcatheter aortic valve replacement (TAVR), aortic valve calcium score (AVCS), paravalvular leak (PVL), balloon-expandable valve (BEV), self-expanding valve (SEV)

## Abstract

**Background:** Aortic valve calcium score (AVCS) is associated with an increased risk of paravalvular leak (PVL) after Transcatheter aortic valve replacement (TAVR). We aimed to evaluate the association between AVCS burden and procedural outcomes, particularly PVL, and to determine whether valve platform performance differs according to calcium burden. **Methods:** This study included patients with severe aortic stenosis undergoing TAVR with balloon-expandable (Sapien platform) or self-expanding (Evolut and Navitor platforms) valves. The primary endpoint was the rate of any and moderate or greater PVLs according to AVCS burden (<3000 vs. ≥3000 AU). Valve platform comparisons were performed within each calcium stratum, and additional analyses evaluated PVLs in contemporary-generation valves. **Results:** A total of 4483 patients were included, of whom 1134 had an AVCS ≥ 3000 AU, and 3349 had an AVCS < 3000 AU. Any type of PVL occurred more frequently in the AVCS ≥ 3000 group (59% vs. 54%; *p* = 0.010), as did moderate or greater PVLs (5% vs. 3%; *p* = 0.011). In multivariable analysis, both SEV implantation and AVCS ≥ 3000 AU were independent predictors of moderate or greater PVL, while the interaction between AVCS burden and valve platform was not significant. When restricted to contemporary-generation valves, PVL rates were lower overall but remained higher in patients with an AVCS ≥ 3000 AU (4.4% vs. 2.4%; *p* = 0.001), with smaller differences between valve platforms. **Conclusions:** A higher AVCS is associated with increased PVLs after TAVR. BEVs showed lower rates of moderate or greater PVLs than SEVs, although no statistically significant interaction between AVCS burden and valve platform was observed. With contemporary-generation valves, overall PVL rates were reduced, and platform differences were attenuated.

## 1. Introduction

Transcatheter aortic valve replacement (TAVR) has become a cornerstone therapy in the treatment of severe aortic stenosis (AS), having evolved into an established and life-saving therapeutic option [1,2,3]. Over the past decade, its global adoption has expanded substantially, driven by progressively broadened clinical indications and continuous refinements in device design and procedural techniques [4,5]. Aortic valve calcium scoring by cardiac computed tomography (CT) is a validated, guideline-endorsed, flow-independent tool for the diagnosis and risk stratification of AS [6,7,8,9]. Quantified in Agatston units (AU), aortic valve calcification provides independent and incremental prognostic value beyond echocardiographic parameters and is strongly associated with adverse outcomes. Sex-specific thresholds reliably identify severe disease and predict disease progression and mortality [10,11]. Aortic valve calcium burden has been consistently associated with an increased risk of paravalvular leak (PVL) following TAVR, particularly with self-expanding valves (SEVs). However, ongoing advancements in device design, including the introduction of external sealing skirts, have markedly reduced PVL rates across both valve platforms in the contemporary era [12,13,14,15,16]. PVLs remain one of the most important procedural complications after TAVR, as moderate or greater PVLs have been consistently associated with increased mortality and adverse clinical outcomes, including heart failure hospitalization. In addition, a significant PVL may lead to persistent symptoms and impaired ventricular remodeling and may increase the need for repeat interventions, including valve-in-valve procedures or surgical correction [17,18].

The aim of this study was to evaluate the association between aortic valve calcium burden and procedural outcomes, with particular emphasis on PVLs, and to determine whether valve platform performance (balloon-expandable versus self-expanding) differs according to calcium burden.

## 2. Methods

We conducted a retrospective single-center analysis using data from the FRANCE TAVR registry at Clinique Pasteur (Toulouse, France). Consecutive patients undergoing TAVR for severe AS between 2016 and 2024 were included. Procedures were performed using balloon-expandable valves (BEVs) (Edwards Sapien XT, Sapien 3, and Sapien 3 Ultra) or SEVs (Medtronic Evolut R, Evolut PRO, Evolut PRO+, Evolut FX, and Navitor). Clinical decisions were determined by a multidisciplinary Heart Team in line with current European Society of Cardiology guidelines for the management of valvular heart disease [1]. Given the retrospective nature of the study and the use of anonymized registry data, the requirement for informed consent was waived in accordance with national regulations.

The primary endpoint of the study was the rate of any and moderate or greater PVLs following TAVR according to aortic valve calcium score (AVCS) burden (<3000 vs. ≥3000 AU). AVCSs were assessed using preprocedural non-contrast cardiac computed tomography and quantified in Agatston units according to standard methodology. CT acquisition was performed according to routine institutional protocols, and calcium scoring was conducted using dedicated software. AVCS measurements were derived retrospectively from clinically acquired scans and measurements were performed as part of routine clinical assessment by experienced operators in cardiac CT at our institution. Given the retrospective nature of the study, AVCS measurements were not systematically performed blinded to clinical outcomes. PVL assessment by transthoracic echocardiography (TTE) was performed by experienced cardiologists specialized in echocardiography at our institution, each with more than 10 years of experience in structural heart disease imaging, including TAVR and transcatheter mitral and tricuspid interventions, and was graded using a standardized multiparametric echocardiographic approach in accordance with the Valve Academic Research Consortium-2 (VARC-2) definitions. Secondary endpoints included in-hospital and 1-year mortality, periprocedural stroke, new permanent pacemaker implantation, major vascular and bleeding complications, and procedural outcomes, including device success, pre- and post-dilatation, valve repositioning, need for a second valve, and coronary obstruction. Clinical and procedural outcomes were adjudicated in accordance with the VARC-2 criteria.

In addition, we performed valve platform comparisons (BEVs versus SEVs) within each calcium stratum and assessed moderate or greater PVLs according to calcium burden and valve platform in contemporary-generation transcatheter valves (Sapien 3, Sapien 3 Ultra, Evolut PRO, Evolut PRO+, Evolut FX, and Navitor). Finally, multivariable analysis was performed to identify independent predictors of moderate or greater PVLs.

## 3. Statistical Analysis

Categorical data are expressed as counts and percentages and were analyzed using the Pearson chi-square test or Fisher’s exact test, as appropriate. The distribution of continuous variables was evaluated for normality. Variables with a normal distribution are presented as mean ± standard deviation and were compared using an unpaired two-sided Student’s *t*-test. Non-normally distributed variables are reported as medians with interquartile ranges and were compared using the Mann–Whitney U test.

Univariable and multivariable logistic regression analyses were performed to identify factors associated with moderate or greater PVLs. Variables included in the multivariable model were age, sex, left ventricular ejection fraction (LVEF), aortic valve area (AVA), EuroSCORE II, valve platform (BEV vs. SEV), AVCS burden (≥3000 vs. <3000 AU), and the interaction term between AVCS burden and valve platform. A sensitivity analysis was performed to evaluate the primary outcomes in patients treated with contemporary-generation TAVR valves.

All statistical analyses were conducted using two-sided tests, with a *p*-value < 0.05 considered indicative of statistical significance. Statistical analyses were performed using SPSS software (version 31.0.0.0; IBM Corp., Armonk, NY, USA).

## 4. Results

A total of 4483 patients were included, of whom 1134 had an AVCS ≥ 3000 AU, and 3349 had AVCS < 3000 AU. Baseline characteristics are summarized in Table 1. The median AVCS was 7477 AU (interquartile range [IQR] 4527–10,429) in the high-calcium group and 1007 AU (IQR 654–1540) in the low-calcium group. The mean age was 84 ± 6 years in the high-calcium group and 83 ± 6 years in the low-calcium group. Overall, baseline clinical characteristics were largely comparable between groups. Hypertension and diabetes mellitus were slightly more frequent in the AVCS < 3000 group (78% vs. 76%, *p* = 0.020 and 24% vs. 20%, *p* = 0.002, respectively). The mean aortic valve area was 0.76 ± 0.18 cm^2^ in the high-calcium group and 0.79 ± 0.19 cm^2^ in the low-calcium group, with corresponding mean gradients of 48 ± 14 mmHg and 46 ± 13 mmHg. Surgical risk scores were similar between groups.

### 4.1. Primary and Secondary Outcomes

**Figure 1:**
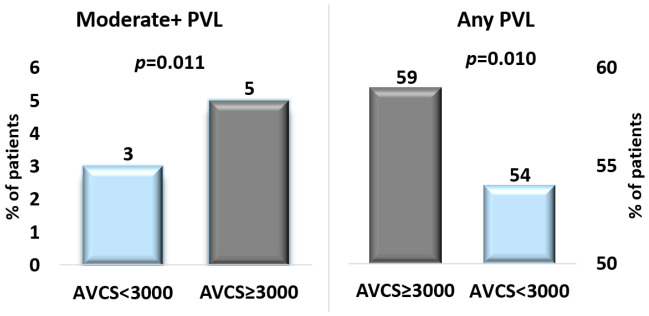
Paravalvular Leak According to Aortic Valve Calcium Burden.

Procedural and clinical outcomes are summarized in Table 2. In the AVCS ≥ 3000 group, 779 patients (69%) received a SEV; in contrast, this figure was 2251 patients (67%) in the AVCS < 3000 group (*p* = 0.357). The vast majority of procedures were performed via a transfemoral approach (98% in both groups). Device success was achieved in 3127 patients (93%) in the AVCS < 3000 group and 1037 patients (91%) in the AVCS ≥ 3000 group (*p* = 0.029). Any PVLs occurred in 667 patients (59%) with an AVCS ≥ 3000, while they occurred in 1822 patients (54%) with an AVCS < 3000 (*p* = 0.010). Moderate or greater PVL was observed in 56 patients (5%) in the high-calcium group and 110 patients (3%) in the low-calcium group (*p* = 0.011) (Figure 1). No significant differences were observed in in-hospital mortality (0.6% vs. 0.3%, *p* = 0.317) or 1-year mortality (6% in both groups). Periprocedural stroke, major vascular complications, and major bleeding were infrequent overall but occurred slightly more frequently in patients with an AVCS ≥ 3000 (periprocedural stroke: 2% vs. 1%, *p* = 0.012; major vascular complications: 3% vs. 2%, *p* = 0.025; major bleeding: 4% vs. 3%, *p* = 0.016). Patients with a higher calcium burden required more post-dilatation (25% vs. 20%, *p* = 0.001), whereas rates of pre-dilatation, valve repositioning, need for a second valve, and coronary obstruction were similar between groups. Procedure duration (47 ± 17 vs. 45 ± 18 min, *p* = 0.015) and contrast volume (85 ± 36 vs. 81 ± 35 mL, *p* = 0.001) were modestly higher in the AVCS ≥ 3000 group.

In multivariable logistic regression analysis for moderate or greater PVLs, both valve platform and aortic valve calcium burden were identified as independent predictors (Table 3). Balloon-expandable valve use (BEVs vs. SEVs) was associated with a lower risk of moderate or greater PVLs (OR 0.453; 95% CI, 0.298–0.689; *p* < 0.001), whereas AVCS ≥ 3000 AU was independently associated with an increased risk (OR 1.429; 95% CI, 1.011–2.020; *p* = 0.043). The interaction term between AVCS burden and valve platform was not statistically significant (OR 2.040; 95% CI, 0.880–4.731; *p* = 0.10), indicating there was no significant effect modification between calcium burden and valve type.

### 4.2. Valve Platform Comparison Within Each Calcium Stratum

**Figure 2:**
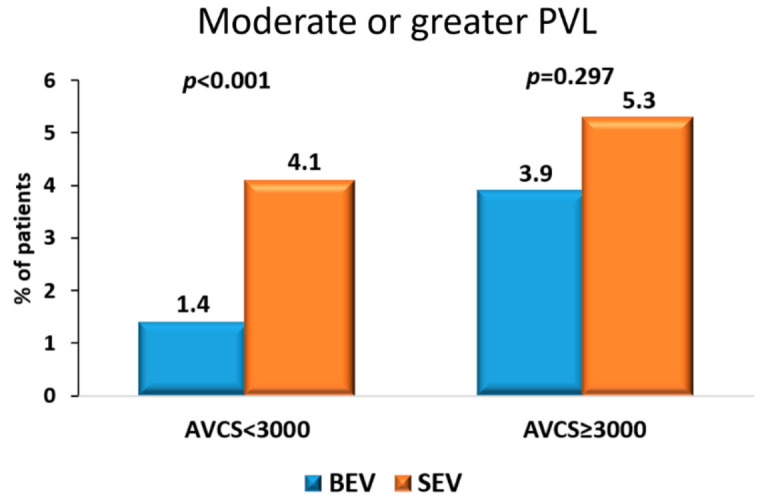
Moderate or Greater Paravalvular Leak According to Aortic Valve Calcium Burden and Valve Platform.

Among patients with an AVCS ≥ 3000 AU, 355 received a BEV and 779 received a SEV (Table 4A, Figure 2). Median calcium scores were similar between groups (7437 [IQR 4264–11,479] AU in the BEV group vs. 7502 [IQR 4632–10,090] AU in the SEV group; *p* = 0.380). Any PVL was significantly more frequent in the SEV group (61% vs. 52%; *p* = 0.002), whereas moderate or greater PVLs was numerically higher with SEVs but did not reach statistical significance (5.3% vs. 3.9%; *p* = 0.297). Rates of post-dilatation (30% vs. 13%) and valve repositioning (40% vs. 1%) were significantly higher in the SEV group (*p* < 0.001 for both). There were no significant differences in periprocedural stroke, in-hospital mortality, pre-dilatation, or the need for a second valve between platforms.

Among patients with an AVCS < 3000 AU, 1098 received BEV and 2251 received SEV (Table 4B, Figure 2). Median calcium score was slightly higher in the BEV group (1093 [IQR 702–1612] AU vs. 976 [IQR 634–1493] AU; *p* = 0.001). Both any PVL (58% vs. 48%; *p* < 0.001) and moderate or greater PVLs (4.1% vs. 1.4%; *p* < 0.001) were significantly more frequent in the SEV group compared with the BEV group. Additionally, rates of pre-dilatation (14% vs. 8%), post-dilatation (25% vs. 12%), and valve repositioning (39% vs. 2%) were higher in the SEV group (*p* < 0.001 for all). No significant differences were observed in periprocedural stroke, in-hospital mortality, or the need for a second valve between platforms.

### 4.3. Moderate or Greater PVL in Contemporary Generation Transcatheter Valves

When restricting the analysis to contemporary-generation transcatheter valves (SAPIEN 3, SAPIEN 3 Ultra, Evolut PRO, Evolut PRO+, Evolut FX, and Navitor), the overall rate of moderate or greater PVL was lower compared with the full cohort, yet remained significantly higher in patients with an AVCS ≥ 3000 AU (4.4% vs. 2.4%; *p* = 0.001) (Table 5A).

Among patients with an AVCS ≥ 3000 AU, moderate or greater PVL was numerically higher in the SEV group compared with the BEV group (4.9% vs. 4.0%), although this difference was not statistically significant (*p* = 0.521) (Table 5B). Among patients with an AVCS < 3000 AU, moderate or greater PVLs remained significantly more frequent with SEVs compared with BEVs (3.1% vs. 1.4%; *p* = 0.005), although the absolute difference between platforms was smaller than in the overall cohort (Table 5C).

## 5. Discussion

In this large cohort of TAVR patients, a higher AVCS was associated with increased rates of PVLs and greater procedural complexity. BEVs were associated with lower rates of moderate or greater PVLs compared with SEVs; however, this effect should be interpreted in the context of a non-randomized comparison and potential residual confounding, and may be influenced by factors such as calcium burden, device generation, and procedural strategy. Importantly, no statistically significant interaction was observed between AVCS burden and valve platform, suggesting that a differential effect across strata was not demonstrated, although numerical differences between subgroups were present and should be interpreted with caution. No differences were observed in in-hospital or 1-year mortality between patients with an AVCS ≥ 3000 and those with lower AVCS. When the analysis was restricted to contemporary-generation valves, overall PVL rates were lower; however, higher AVCSs remained associated with an increased risk of PVL, and platform-related differences persisted, albeit with a smaller magnitude. Rates of pre- and post-dilatation, as well as valve repositioning, were higher with SEVs. These findings suggest that the attenuation of differences with contemporary-generation valves and the higher rates of post-dilatation and valve repositioning observed with SEVs may reflect the influence of procedural factors and device design on the relationship between valve platform and PVL.

The relatively high rate of ‘any PVL’ observed in this study likely reflects the inclusion of trace and mild regurgitation, which is common after TAVR and is generally of limited clinical significance. Accordingly, moderate-or-greater PVL was considered the clinically relevant endpoint and represents the primary focus of this analysis.

Several mechanisms may explain the association between increased AVCS and the risk of PVL following TAVR. A higher calcium burden may reduce annular compliance and limit the ability of the prosthesis to achieve full and symmetric expansion. In addition, bulky or asymmetric calcification may lead to incomplete apposition between the transcatheter valve and the native annulus, resulting in paravalvular regurgitation. Calcification extending into the LVOT or subannular region may further interfere with prosthesis sealing and positioning. These biomechanical interactions between the prosthesis and the native valve complex likely play a central role in the development of PVL in heavily calcified valves.

Several studies using preprocedural cardiac CT have evaluated the impact of AVCS on the risk of PVL after TAVR. Larroche et al. [12] evaluated the impact of AVCS burden on TAVR outcomes in a cohort of 352 patients and showed that AVCS measured on preprocedural contrast-enhanced CT was associated with lower device success and higher rates of PVL, but not with 30-day major adverse cardiovascular events (MACE). Another study [19] investigated the role of AVCS in predicting PVL after TAVR in a cohort of 965 patients and demonstrated that higher AVCS independently correlates with the development of PVL and improves risk stratification in patients undergoing TAVR. Tomii et al. [13] evaluated 1345 patients undergoing TAVR with BEV or SEV and demonstrated that BEV implantation was associated with a lower risk of PVL and permanent pacemaker implantation but also a higher risk of annular rupture compared with SEV. Long-term mortality, however, was comparable between the two valve platforms over five years of follow-up.

In recent years, the incidence of PVLs has progressively declined with iterative improvements in TAVR technology and implantation techniques. Newer-generation prostheses incorporate external sealing mechanisms, such as outer pericardial wraps, designed to enhance annular sealing and reduce PVL [15,16]. Akodad et al. [20] evaluated the prognostic impact of AVCS patients undergoing TAVR with first-generation (Sapien XT and CoreValve) and newer-generation valves (Sapien 3 and Evolut R). In a cohort of 346 patients, higher calcium burden was associated with adverse clinical events and PVL in patients treated with first-generation devices. However, this association was attenuated with newer-generation valves, where calcium score was no longer predictive of adverse clinical events. Nevertheless, higher calcium burden remained associated with PVL in patients treated with the self-expanding Evolut R valve. Another study evaluated the impact of AVCS on outcomes after TAVR with the newer-generation BEV Sapien 3 valve and demonstrated low rates of significant PVL irrespective of calcium burden. Furthermore, the severity of aortic valve calcification did not influence mortality or stroke risk following TAVR with the Sapien 3 valve [21]. Taken together, our findings add important data to the existing literature by evaluating the impact of AVCS on PVL in a large real-world TAVR cohort including both BEV and SEV platforms. Higher AVCSs and SEV use remained associated with an increased risk of moderate or greater PVL. At the same time, a lower overall incidence of PVL was observed with newer-generation valves, and the differences between valve platforms became smaller.

It is important to recognize that AVCS represents only one component of aortic valve pathology. AS is increasingly understood as a fibro-calcific disease, and calcium burden alone may not fully capture leaflet stiffness, deformability, or prosthesis–annulus interaction. Recent advances in cardiac CT imaging have enabled differentiation between calcific and fibrotic tissue components. In this context, Grodecki et al. [22] demonstrated that CT angiography can accurately quantify both components with excellent agreement with histology, highlighting that fibrosis constitutes a substantial proportion of valve pathology and may have independent biomechanical implications. These findings suggest that more comprehensive tissue characterization beyond calcium burden alone may further refine risk stratification and procedural planning in TAVR. In addition, the use of a binary AVCS threshold in the present analysis provides a pragmatic and clinically interpretable approach but may oversimplify the relationship between calcium burden and PVL risk. Modeling AVCS as a continuous variable or using more advanced approaches such as spline analysis may better capture potential dose-response relationships. Future studies incorporating ROC or other continuous modeling approaches may help refine optimal AVCS thresholds for predicting PVLs. Furthermore, although sex-specific thresholds are well established for the diagnosis of severe aortic stenosis, their relevance for predicting procedural outcomes such as PVLs remains uncertain and was not addressed in the current analysis.

Our findings have important clinical implications. AVCSs remain an important marker of PVL risk and should continue to play a role in preprocedural planning for TAVR. However, contemporary imaging strategies are increasingly evolving beyond calcium quantification alone toward more advanced three-dimensional CT-based visualization and planning tools, which enable improved anatomical assessment and procedural sizing [23]. Valve platform selection may influence PVL risk, as BEVs were associated with lower rates of moderate or greater PVLs compared with SEVs. Importantly, despite higher PVL rates in patients with elevated AVCS, mortality outcomes were similar, supporting the safety of TAVR in heavily calcified valves. Finally, the lower PVL rates observed with contemporary-generation valves and the smaller differences between valve platforms highlight the impact of ongoing technological improvements and the incorporation of external sealing skirts designed to enhance annular sealing. Future studies should evaluate whether integrating AVCS into procedural planning and valve selection can reduce PVL after TAVR. In addition, the role of advanced imaging, longer-term follow-up, and potential sex-specific differences in calcium burden and PVL risk should be explored to better define the clinical impact of residual PVL in the contemporary era.

## 6. Study Limitations

This study has several limitations that warrant consideration. First, its retrospective observational design is inherently subject to residual confounding and potential selection bias. Second, despite the relatively large cohort, the single-center design may limit the generalizability of the findings to other settings with differing patient populations, levels of operator expertise, and procedural approaches. Third, PVL assessment was based on TTE performed during the index hospitalization prior to discharge, so it may be subject to interobserver variability and less sensitive than transesophageal echocardiography or quantitative assessment methods. Fourth, the present analysis relied on global AVCSs and did not account for the spatial distribution of calcium within the aortic root. Regional features such as LVOT, commissural, annular, or subannular calcification, as well as calcium asymmetry, may have important implications for valve expansion and PVL, and they were not captured in this analysis. In addition, the comparison between valve platforms was not randomized, and residual confounding due to unmeasured anatomical and procedural factors, such as annular dimensions, bicuspid anatomy, valve-sizing strategy, implantation depth, and treatment era, cannot be excluded. Therefore, differences observed between BEVs and SEVs should be interpreted as associative rather than causal. Fifth, formal interobserver variability assessment for AVCS measurements was not performed. Finally, although multivariable analysis was performed and adjusted for relevant clinical and echocardiographic variables, residual confounding from unmeasured anatomical or procedural factors cannot be excluded.

In conclusion, a higher AVCS is associated with greater PVLs following TAVR. BEVs were associated with lower rates of moderate or greater PVLs compared with SEVs, although no statistically significant interaction between AVCS burden and valve platform was observed. In contemporary-generation valves, overall PVL rates were attenuated, and the differences between valve platforms were smaller. These findings suggest that AVCSs may serve as a simple and clinically applicable tool for preprocedural risk stratification, potentially guiding valve platform selection and procedural planning for patients undergoing TAVR. However, such an approach requires validation in larger, prospective cohorts before it can be incorporated into routine clinical practice.

## 7. Clinical Perspectives

- A higher aortic valve calcium score (AVCS) is associated with an increased risk of paravalvular leak (PVL) after TAVR.
- Balloon-expandable valves are associated with lower rates of moderate or greater PVLs compared with self-expanding valves across calcium strata, with no significant interaction between AVCS burden and valve platform.
- With contemporary-generation valves, overall PVL rates are lower, and differences between valve platforms are attenuated.

## Figures and Tables

**Table 1:** Baseline characteristics.

Characteristic	AVCS ≥ 3000 AU	AVCS < 3000 AU	*p*-Value
*n*	1134	3349	
Age, years (mean ± SD)	84 ± 6	83 ± 6	<0.001
Gender, male *n* (%)	563 (50)	1884 (56)	<0.001
Calcium Score, AU, median (Q1, Q3)	7477 (4527, 10,429)	1007 (654, 1540)	<0.001
Cardiovascular Comorbidities			
Dyslipidemia, *n* (%)	538 (47)	1560 (46)	0.615
Hypertension, *n* (%)	857 (76)	2642 (78)	0.020
Diabetes mellitus, *n* (%)	226 (20)	815 (24)	0.002
Coronary artery disease, *n* (%)	494 (44)	1438 (43)	0.714
Prior PCI, *n* (%)	359 (32)	996 (30)	0.224
Prior CABG, *n* (%)	59 (5)	184 (5)	0.708
Prior CVA/TIA, *n* (%)	107 (9)	282 (8)	0.295
PVD, *n* (%)	134 (12)	426 (13)	0.424
Atrial Fibrillation, *n* (%)	212 (19)	697 (21)	0.125
Baseline TTE Data			
LVEF%, (mean ± SD)	58 ± 11	59 ± 11	0.773
AVA, cm^2^ (mean ± SD)	0.76 ± 0.18	0.79 ± 0.19	<0.001
Mean Aortic gradient, mmHg, (mean ± SD)	48 ± 14	46 ± 13	<0.001
Surgical Risk			
Euroscore II, median (Q1, Q3)	3.4 (2.1, 5.5)	3.2 (2.0, 5.4)	0.254
STS Score, median (Q1, Q3)	3.6 (2.4, 5.4)	3.7 (2.4, 5.5)	0.272

AVCS = Aortic valve calcium score; AU = Agatston units; PCI = Percutaneous coronary intervention; CABG = Coronary artery bypass graft; CVA = Cerebrovascular Accident; TIA = Transient ischemic attack; PVD = Peripheral vascular disease; LVEF = Left ventricular ejection fraction; AVA = Aortic valve area.

**Table 2:** Procedural and clinical outcomes.

Characteristic	AVCS ≥ 3000 AU	AVCS < 3000 AU	*p*-Value
*n*	1134	3349	
SEV, *n* (%)	779 (69)	2251 (67)	0.357
Transfemoral approach, *n* (%)	1115 (98)	3279 (98)	0.387
Device success *, *n* (%)	1037 (91)	3127 (93)	0.029
Primary outcomes			
Any PVL (Echo), *n* (%)	667 (59)	1822 (54)	0.010
Moderate or greater PVL (Echo), *n* (%)	56 (5)	110 (3)	0.011
Secondary outcomes			
Pre-dilatation, *n* (%)	161 (14)	414 (12)	0.110
Post-dilatation, *n* (%)	288 (25)	697 (20)	0.001
Valve repositioning, *n* (%)	318 (28)	894 (26)	0.377
Need for second valve, *n* (%)	10 (1)	28 (1)	0.884
Coronary obstruction, *n* (%)	5 (0.5)	6 (0.1)	0.124
Echo mean gradient post-TAVR, (mean ± SD)	9 ± 4	10 ± 4	0.002
Procedure Duration, minutes (mean ± SD)	47 ± 17	45 ± 18	0.015
Contrast volume, mL (mean ± SD)	85 ± 36	81 ± 35	0.001
PeriProcedural Stroke, *n* (%)	20 (2)	29 (1)	0.012
Permanent Pacemaker, *n* (%)	155 (14)	522 (15)	0.065
Major vascular complication, *n* (%)	34 (3)	63 (2)	0.025
Major bleeding, *n* (%)	45 (4)	86 (3)	0.016
In-hospital mortality, *n* (%)	7 (0.6)	13 (0.3)	0.317
1 year mortality, *n* (%)	67 (6)	192 (6)	0.834

AVCS = Aortic valve calcium score; AU = Agatston units; SEV = Self-expanding valve; PVL = Paravalvular leak. * Defined as absence of procedural mortality, correct position of a single prosthetic valve with a mean aortic gradient < 20 mmHg or peak velocity < 3 m/s and no moderate or severe prosthetic aortic valve regurgitation.

**Table 3:** Multivariable analysis for moderate or greater paravalvular leaks.

	Univariable OR (95% CI)	*p*-Value	Multivariable OR (95% CI) *	*p*-Value
Age	1.016 (95% CI, 0.989–1.044)	0.243	-	
Gender, male	0.891 (95% CI, 0.653–1.215)	0.464	-	
LVEF%	0.992 (95% CI, 0.979–1.004)	0.200	-	
AVA, cm^2^	0.503 (95% CI, 0.207–1.221)	0.129	-	
Euroscore II	1.006 (95% CI, 0.973–1.041)	0.721	-	
Valve type (BEV vs. SEV)	0.449 (95% CI, 0.301–0.670)	<0.001	0.453 (95% CI, 0.298–0.689)	<0.001
AVCS ≥ 3000 AU	1.530 (95% CI, 1.101–2.126)	0.011	1.429 (95% CI, 1.011–2.020)	0.043
Interaction *: AVCS × Valve type	1.074 (95% CI, 0.614–1.877)	0.802	2.040 (95% CI, 0.880–4.731)	0.100

LVEF = Left ventricular ejection fraction; AVA = Aortic valve area; AU = Agatston units; BEV = Balloon-expandable valve; SEV = Self-expanding valves; AVCS = Aortic valve calcium score. * Interaction term represents the product of AVCS ≥ 3000 AU and valve platform (BEV vs. SEV).

**Table 4:** Valve Platform Comparison Within Each Calcium Stratum. (**A**) AVCS ≥ 3000 AU. (**B**) AVCS < 3000 AU.

(**A**)			
**Characteristic**	**BEV**	**SEV**	***p*-Value**
*n* (1134)	355	779	
Calcium Score, AU, median (Q1, Q3)	7437 (4264, 11,479)	7502 (4632, 10,090)	0.380
Main outcomes			
Any PVL (Echo), *n* (%)	185 (52)	482 (61)	0.002
Moderate or greater PVL (Echo), *n* (%)	14 (3.9)	42 (5.3)	0.297
PeriProcedural Stroke, *n* (%)	6 (1.6)	14 (1.7)	0.899
In-hospital mortality, *n* (%)	1 (0.8)	4 (0.5)	0.684
Procedural data			
Pre-dilatation, *n* (%)	48 (13)	113 (15)	0.660
Post-dilatation, *n* (%)	48 (13)	240 (30)	<0.001
Valve repositioning, *n* (%)	5 (1)	313 (40)	<0.001
Need for second valve, *n* (%)	1 (0.3)	9 (1.1)	0.186
(**B**)			
**Characteristic**	**BEV**	**SEV**	***p*** **-Value**
*n*	1098	2251	
Calcium Score, AU, median (Q1, Q3)	1093 (702, 1612)	976 (634, 1493)	0.001
Main outcomes			
Any PVL (Echo), *n* (%)	523 (48)	1299 (58)	<0.001
Moderate or greater PVL (Echo), *n* (%)	16 (1.4)	94 (4.1)	<0.001
PerProcedural Stroke, *n* (%)	8 (0.7)	21 (0.9)	0.549
In-hospital mortality, *n* (%)	2 (0.2)	11 (0.4)	0.073
Procedural data			
Pre-dilatation, *n* (%)	85 (8)	329 (14)	<0.001
Post-dilatation, *n* (%)	136 (12)	561 (25)	<0.001
Valve repositioning, *n* (%)	18 (2)	876 (39)	<0.001
Need for second valve, *n* (%)	6 (0.5)	22 (0.9)	0.199

AVCS = Aortic valve calcium score; AU = Agatston units; PVL = Paravalvular leak.

**Table 5:** Moderate or Greater Paravalvular Leak According to Aortic Valve Calcium Burden and Valve Platform in Contemporary-Generation Transcatheter Valves (SAPIEN 3, SAPIEN 3 Ultra, Evolut PRO, Evolut PRO+, Evolut FX, and Navitor valves). (**A**) Overall patients according to AVCS. (**B**) AVCS ≥ 3000 AU. (**C**) AVCS < 3000 AU.

(**A**)			
**Characteristic**	**AVCS ≥ 3000 AU**	**AVCS < 3000 AU**	***p*-Value**
*n* (3416)	915	2501	
Moderate or greater PVL (Echo), *n* (%)	41 (4.4)	61 (2.4)	0.001
(**B**)			
**Characteristic**	**BEV**	**SEV**	***p*** **-Value**
*n*	348	567	
Moderate or greater PVL (Echo), *n* (%)	14 (4.0)	28 (4.9)	0.521
(**C**)			
**Characteristic**	**BEV**	**SEV**	***p*** **-Value**
*n*	1092	1409	
Moderate or greater PVL (Echo), *n* (%)	16 (1.4)	45 (3.1)	0.005

AVCS = Aortic valve calcium score; AU = Agatston units; PVL = Paravalvular leak; BEV = Balloon-expandable valve; SEV = Self-expanding valve.

## Data Availability

The data supporting the findings of this study are available from the corresponding author upon reasonable request.

## Abbreviations

TAVR	Transcatheter aortic valve replacement.
AS	Aortic stenosis.
AVCS	Aortic valve calcium score.
PVL	paravalvular leak.
BEV	Balloon-expandable valve.
SEV	Self-expanding valve.
AU	Agatston units.
TTE	Transthoracic echocardiography.

## References

[1] Vahanian A., Beyersdorf F., Praz F., Milojevic M., Baldus S., Bauersachs J., Capodanno D., Conradi L., De Bonis M., De Paulis R. (2022). 2021 ESC/EACTS Guidelines for the management of valvular heart disease: Developed by the Task Force for the management of valvular heart disease of the European Society of Cardiology (ESC) and the European Association for Cardio-Thoracic Surgery (EACTS). Rev. Española Cardiol..

[2] Otto C.M., Nishimura R.A., Bonow R.O., Carabello B.A., Erwin J.P., Gentile F., Jneid H., Krieger E.V., Mack M., McLeod C. (2021). 2020 ACC/AHA Guideline for the Management of Patients with Valvular Heart Disease: A Report of the American College of Cardiology/American Heart Association Joint Committee on Clinical Practice Guidelines. Circulation.

[3] Praz F., Beyersdorf F., Haugaa K., Prendergast B. (2024). Valvular heart disease: From mechanisms to management. Lancet.

[4] Goldsweig A.M., Thourani V.H. (2022). Decreasing Prices but Increasing Demand for Transcatheter Aortic Valve Replacement. Circ. Cardiovasc. Interv..

[5] Nguyen V., Willner N., Eltchaninoff H., Burwash I.G., Michel M., Durand E., Gilard M., Dindorf C., Iung B., Cribier A. (2022). Trends in aortic valve replacement for aortic stenosis: A French nationwide study. Eur. Heart J..

[6] Pawade T.A., Newby D.E., Dweck M.R. (2015). Calcification in Aortic Stenosis: The Skeleton Key. J. Am. Coll. Cardiol..

[7] Doris M.K., Everett R.J., Shun-Shin M., Clavel M.-A., Dweck M.R. (2019). The Role of Imaging in Measuring Disease Progression and Assessing Novel Therapies in Aortic Stenosis. JACC Cardiovasc. Imaging.

[8] Clavel M.-A., Burwash I.G., Pibarot P. (2017). Cardiac Imaging for Assessing Low-Gradient Severe Aortic Stenosis. JACC Cardiovasc. Imaging.

[9] Wanchaitanawong W., Kanjanavanit R., Srisuwan T., Wongcharoen W., Phrommintikul A. (2023). Diagnostic role of aortic valve calcium scoring in various etiologies of aortic stenosis. Sci. Rep..

[10] Pawade T., Clavel M.-A., Tribouilloy C., Dreyfus J., Mathieu T., Tastet L., Renard C., Gun M., Jenkins W.S.A., Macron L. (2018). Computed Tomography Aortic Valve Calcium Scoring in Patients with Aortic Stenosis. Circ. Cardiovasc. Imaging.

[11] Desai M.Y., Braunwald E. (2025). The Pathophysiologic Basis and Management of Calcific Aortic Valve Stenosis: JACC State-of-the-Art Review. J. Am. Coll. Cardiol..

[12] Larroche J., Panh L., Lhermusier T., Bataille V., Marachet M.A., Chollet T., Petermann A., Bouisset F., Boudou N., Marcheix B. (2020). Impact of aortic valve calcification severity on device success after transcatheter aortic valve replacement. Int. J. Cardiovasc. Imaging.

[13] Tomii D., Alaour B., Heg D., Okuno T., Nakase M., Samim D., Praz F., Lanz J., Stortecky S., Reineke D. (2026). Self-expanding versus balloon-expandable transcatheter heart valves in patients with excessive aortic valve cusp calcification. Am. Heart J..

[14] Kim W.-K., Blumenstein J., Liebetrau C., Rolf A., Gaede L., Van Linden A., Arsalan M., Doss M., Tijssen J.G.P., Hamm C.W. (2017). Comparison of outcomes using balloon-expandable versus self-expanding transcatheter prostheses according to the extent of aortic valve calcification. Clin. Res. Cardiol..

[15] Welle G.A., El-Sabawi B., Thaden J.J., Greason K.L., Klarich K.W., Nkomo V.T., Alkhouli M.A., Guerrero M.E., Crestanello J.A., Holmes D.R. (2021). Effect of a fourth-generation transcatheter valve enhanced skirt on paravalvular leak. Catheter. Cardiovasc. Interv..

[16] Matta A., Regueiro A., Urena M., Nombela-Franco L., Riche M., Rodriguez-Gabella T., Amat-Santos I., Chamandi C., Akiki T., Gabani R. (2023). Comparison of Paravalvular Leak in SAPIEN 3 and EVOLUT PRO Valves in Transcatheter Aortic Valve Replacement: A Multicenter Registry. Am. J. Cardiol..

[17] Sinning J.-M., Vasa-Nicotera M., Chin D., Hammerstingl C., Ghanem A., Bence J., Kovac J., Grube E., Nickenig G., Werner N. (2013). Evaluation and management of paravalvular aortic regurgitation after transcatheter aortic valve replacement. J. Am. Coll. Cardiol..

[18] Laakso T., Laine M., Moriyama N., Dahlbacka S., Airaksinen J., Virtanen M., Husso A., Tauriainen T., Niemelä M., Mäkikallio T. (2020). Impact of paravalvular regurgitation on the mid-term outcome after transcatheter and surgical aortic valve replacement. Eur. J. Cardiothorac. Surg..

[19] Kofler M., Meyer A., Schwartz J., Sündermann S., Penkalla A., Solowjowa N., Klein C., Unbehaun A., Falk V., Kempfert J. (2021). A new calcium score to predict paravalvular leak in transcatheter aortic valve implantation. Eur. J. Cardiothorac. Surg..

[20] Akodad M., Lattuca B., Agullo A., Macia J.C., Gandet T., Marin G., Iemmi A., Vernhet H., Schmutz L., Nagot N. (2018). Prognostic Impact of Calcium Score after Transcatheter Aortic Valve Implantation Performed with New Generation Prosthesis. Am. J. Cardiol..

[21] Guimarães L., Ferreira-Neto A.N., Urena M., Nombela-Franco L., Wintzer-Wehekind J., Levesque M.-H., Himbert D., Fischer Q., Armijo G., Vera R. (2020). Transcatheter aortic valve replacement with the balloon-expandable SAPIEN 3 valve: Impact of calcium score on valve performance and clinical outcomes. Int. J. Cardiol..

[22] Grodecki K., Olasińska-Wiśniewska A., Cyran A., Urbanowicz T., Kwieciński J., Geers J., Tamarappoo B.K., Perek B., Gocoł R., Nawara-Skipirzepa J. (2024). Quantification of Aortic Valve Fibrotic and Calcific Tissue from CTA: Prospective Comparison with Histology. Radiology.

[23] Bonanni M., Russo G., De Siati M., Tomao F., Massaro G., Benedetto D., Longoni M., Matteucci A., Maffi V., Mariano E.G. (2024). Holographic mixed reality for planning transcatheter aortic valve replacement. Int. J. Cardiol..

